# Performance in dynamic movement tasks and occurrence of low back pain in youth floorball and basketball players

**DOI:** 10.1186/s12891-020-03376-1

**Published:** 2020-06-05

**Authors:** M. K. Rossi, K. Pasanen, A. Heinonen, S. Äyrämö, A. M. Räisänen, M. Leppänen, G. Myklebust, T. Vasankari, P. Kannus, J. Parkkari

**Affiliations:** 1 Tampere Research Center of Sports Medicine, UKK Institute, 33501 Tampere, Finland; 2 Faculty of Sport and Health Sciences, University of Jyväskylä, Jyväskylä, Finland; 3 Sport Injury Prevention Research Centre, Faculty of Kinesiology, University of Calgary, Calgary, Canada; 4 Alberta Children’s Hospital Research Institute, University of Calgary, Calgary, Canada; 5 McCaig Institute for Bone and Joint Health, University of Calgary, Calgary, Canada; 6 Faculty of Information Technology, University of Jyväskylä, Jyväskylä, Finland; 7 Oslo Sports Trauma Research Center, Department of Sports Sciences, Norwegian School of Sport Sciences, Oslo, Norway; 8 Department of Orthopedics & Traumatology, Tampere University Hospital, Tampere, Finland; 9 Tampere University Hospital, Tampere, Finland

**Keywords:** Low back pain, Lumbar spine, Team sports, Youth athletes, Risk factors

## Abstract

**Background:**

Prospective studies investigating risk factors for low back pain (LBP) in youth athletes are limited. The aim of this prospective study was to investigate the association between hip-pelvic kinematics and vertical ground reaction force (vGRF) during landing tasks and LBP in youth floorball and basketball players.

**Methods:**

Three-hundred-and-eighty-three Finnish youth female and male floorball and basketball players (mean age 15.7 ± 1.8) participated and were followed up on for 3 years. At the beginning of every study year the players were tested with a single-leg vertical drop jump (SLVDJ) and a vertical drop jump (VDJ). Hip-pelvic kinematics, measured as femur-pelvic angle (FPA) during SLVDJ landing, and peak vGRF and side-to-side asymmetry of vGRF during VDJ landing were the investigated risk factors. Individual exposure time and LBP resulting in time-loss were recorded during the follow-up. Cox’s proportional hazard models with mixed effects and time-varying risk factors were used for analysis.

**Results:**

We found an increase in the risk for LBP in players with decreased FPA during SLVDJ landing. There was a small increase in risk for LBP with a one-degree decrease in right leg FPA during SLVDJ landing (HR 1.09, 95% CI 1.02 to 1.17, per one-degree decrease of FPA). Our results showed no significant relationship between risk for LBP and left leg FPA (HR 1.04, 95% CI 0.97 to 1.11, per one-degree decrease of FPA), vGRF (HR 1.83, 95% CI 0.95 to 3.51) or vGRF side-to-side difference (HR 1.22, 95% CI 0.65 to 2.27) during landing tasks.

**Conclusions:**

Our results suggest that there is an association between hip-pelvic kinematics and future LBP. However, we did not find an association between LBP and vGRF. In the future, the association between hip-pelvic kinematics and LBP occurrence should be investigated further with cohort and intervention studies to verify the results from this investigation.

**Level of evidence:**

Prognosis, level 1b.

## Key points

**Findings** Based on the results of this study, peak vGRF is a poor risk factor for LBP in youth team sport players. Hip-pelvic kinematics are associated with increased risk for LBP; smaller angle between the femur and pelvis increases the risk for all LBP and non-traumatic gradual onset LBP.

**Implications** One cannot discriminate players with future LBP based on the femur-pelvic angle during SLVDJ landing alone. The association between hip-pelvic kinematics and other movement patterns, such as trunk kinematics, and risk for LBP in athletes merits further investigations.

**Caution** The data recording and statistical analyses in this study did not take into account the temporal nature of physical abilities during the follow-up nor did it include psychosocial factors. Statistical power might not have been enough to reveal small to moderate associations. The results should be verified by future cohort and intervention studies.

## Background

Back pain is common among youth athletes [1]. Our previous findings show that nearly half of floorball players (45%) and 64% of basketball players have had LBP during the preceding 12 months [2]. Furthermore, lower extremity injuries (LEI) resulting in time loss are common among these players [3]. Association between LEI and back pain has been suggested by previous research [4–6]. It has been speculated that changes in lower extremity function after an injury, or shared risk factors, might explain the association between LEI and LBP and that plausible mechanisms behind this relationship should be investigated [5]. Sports injury studies have investigated the association between LEI and lower extremity kinetics and kinematics, such as ground reaction forces and lower extremity movement patterns, but they have not considered how these factors might contribute to the cause of LBP.

Previous studies investigating intrinsic risk factors for LBP in youth have focused mostly on lower extremity and trunk muscle strength and endurance, flexibility and anthropometric measures [1, 7]. Prospective investigations into association between LBP and movement patterns in youth athletes are scarce [8] and most of the previous studies investigating back pain in athletes have been largely cross-sectional [9].

It has been stated that the trunk, including lumbo–pelvic–hip complex, is the central point of kinetic chains of most sports activities and essential in decreasing back injuries [10]. Furthermore, it has been suggested that for the functional evaluation of the trunk and lumbo–pelvic–hip complex, dynamic hip-pelvic movement patterns should be investigated [10]. Previous research has identified differences between youth athletes with and without LBP on lumbo–pelvic–hip complex movement patterns [11–14] and an association between LBP and frontal plane hip-pelvic movement patterns has been observed in single-leg dynamic tasks in youth cricket players [15] and in adults with LBP [16].

Basketball and floorball (an indoor team ball sport that resembles floor hockey) are sports that include running, sudden direction changes and stops. In addition, basketball players perform lots of jumping and landing [17]. These movements produce large ground reaction forces (GRF) [18, 19] that transfer to the lumbar spine and thus may pre-dispose players to LBP. Yet, to our knowledge, the association between LBP and peak vGRF nor lumbo–pelvic–hip complex movement patterns, using kinematic measures, have not been investigated in youth floorball and basketball players.

The aim of this exploratory prospective study was to investigate if hip-pelvic kinematics, measured as femur-pelvic angle (FPA), and peak vGRF during landing tasks, are associated with LBP incidence in a large cohort of youth basketball and floorball players. The prospective design and consideration of the individual training and game exposure hours adds to the novelty value of this study. The hypotheses were that [1] decreased FPA in frontal plane during single-leg vertical drop jump (SLVDJ) landing and [2] higher or asymmetric peak vGRF during vertical drop jump (VDJ) landing increase the risk for LBP plausibly due to increased load and strain in the lumbo-pelvic area.

## Methods

### Study design and data collection

This study is part of the large Finnish PROFITS study (Predictors of Lower Extremity Injuries in Team Sports) carried out between 2011 and 2015 [20] and the descriptive results regarding LBP have been reported already in previous reports [1, 2]. This study was approved by the Ethics Committee of the Pirkanmaa Hospital District (ETL-code R10169) and carried out in accordance with the Declaration of Helsinki and the guidelines for good scientific practice. Written informed consent was acquired from the participants (and a legal guardian if the player was under 18 years old).

Ten female and male basketball and 10 floorball teams were recruited from six sports clubs in Tampere, Finland. Players older than 21 and younger than 12 at baseline were excluded. Data were collected at baseline in April or May of 2011, 2012, or 2013 as the player entered the study, and at the beginning of each study year in which the player participated. The players were followed prospectively for up to 3 years. Data from all players entering the follow-up were included in the analyses for the time they participated.

The baseline questionnaire covered the following demographics: age, sex, dominant leg, nicotine use, family history of musculoskeletal disorders, and training and playing history during the previous 12 months.

The players’ history of back pain was recorded using the Standardized Nordic questionnaire of musculoskeletal symptoms (modified version for athletes) [21, 22]. History of previous LBP was determined based on the question: How many days have you had LBP during the past 12 months: ‘none’ (recorded as no LBP history), ‘1 to 7 days’, ‘8 to 30 days’, ‘>30 days but not daily’ and ‘daily’ (recorded as a history of LBP). The questionnaire has been validated among adults [23]. The baseline questionnaire was completed during the same day as the baseline tests.

The baseline tests were performed at the UKK Institute over 1 day at the beginning of every follow-up year. The test procedures are outlined in more detail in previous reports [20, 24–29] and Table 1 and only briefly described below. Players with an ongoing injury at the time of the baseline test and players who did not have a valid number of test trials were excluded from the risk factor analyses.

**Table 1:** Description of selected baseline tests and the investigated variables

**SINGLE-LEG VERTICAL DROP JUMP (SLVDJ)**	
**Preparation** Small pieces of sports tape were placed on the left and right side of the upper anterior iliac tubercle (ASIS) and tuberositas tibiae.	
**Equipment** A high-definition digital camera (Sony® Digital HD Video Camera Recorder HXR-NX70E, Sony Corporation, Tokyo, JAPAN).	
**Warm up** No separate warm-up was performed, as the SLVDJ immediately followed a previous test (not included in this study). One practice trial on each leg was allowed.	
**Test performance** During the test the player stood in front of the video camera, on a 10-cm box. Using one leg, the player dropped off the box and landed on one leg. Immediately after landing, the player performed a maximal jump straight up with the same leg. (The test was performed three times.) An overhead goal was used for maximum effort [30] and the test started with the right leg. Trials with jumping, a leg touching the ground or falling/clear loss of balance, were considered invalid. Two valid trials was considered acceptable.	
**Measurements/Calculations** The frontal plane knee and pelvic angles were estimated by a physiotherapist by marking the knee joint centre and ASIS in the still image captured from a video. Joint angles were estimated at the point of maximum knee flexion during initial landing. *Femur-pelvic angle (FPA)* described the angle between the femur and pelvis and was calculated from the intersection of a line created by ASIS and the knee joint centre. A smaller angle indicates increased femur adduction and/or pelvic drop.	
**VERTICAL DROP JUMP (VDJ):**	
**Preparation** A static calibration trial was performed.	
**Equipment** The 3D motion analysis consisted of eight cameras (Vicon T40, Oxford, UK), 16 lower body markers (Plug-In Gait, Vicon, Oxford, UK) and two force plates (AMTI, Watertown, Massachusetts) where data were recorded synchronously at 300 fps and 1500 Hz. A 30-cm box was used.	
**Warm up** Players performed a standardised warm-up (5 min of cycling) before testing. One practice trial was allowed.	
**Test performance** The player stood on the 30-cm box, dropped off the box and landed symmetrically on both feet on the force plates. Immediately after landing the player jumped as high as possible. An overhead goal was used for maximum effort [30] and the player tried to touch the goal with their head. Three valid trials were collected. The trials were accepted if the entire foot landed on the force plate and the markers stayed tightly on the athlete’s skin throughout the task.	
**Measurements/Calculations** Vicon Nexus Plug-in Gait model was used for the analyses. Peak vGRF and vGRF asymmetry were investigated as potential risk factors. Three trials from both legs were averaged and the side with the larger value was chosen for analyses as peak vGRF. Peak vGRF was normalized by bodyweight. The vGRF asymmetry was calculated as the difference between the right and left legs. GRF was filtered using a fourth-order Butterworth filter with cutoff frequencies of 15 Hz and the landing phase was defined as the period when the unfiltered ground reaction force exceeded 20 N.	

**Fig. 1:**
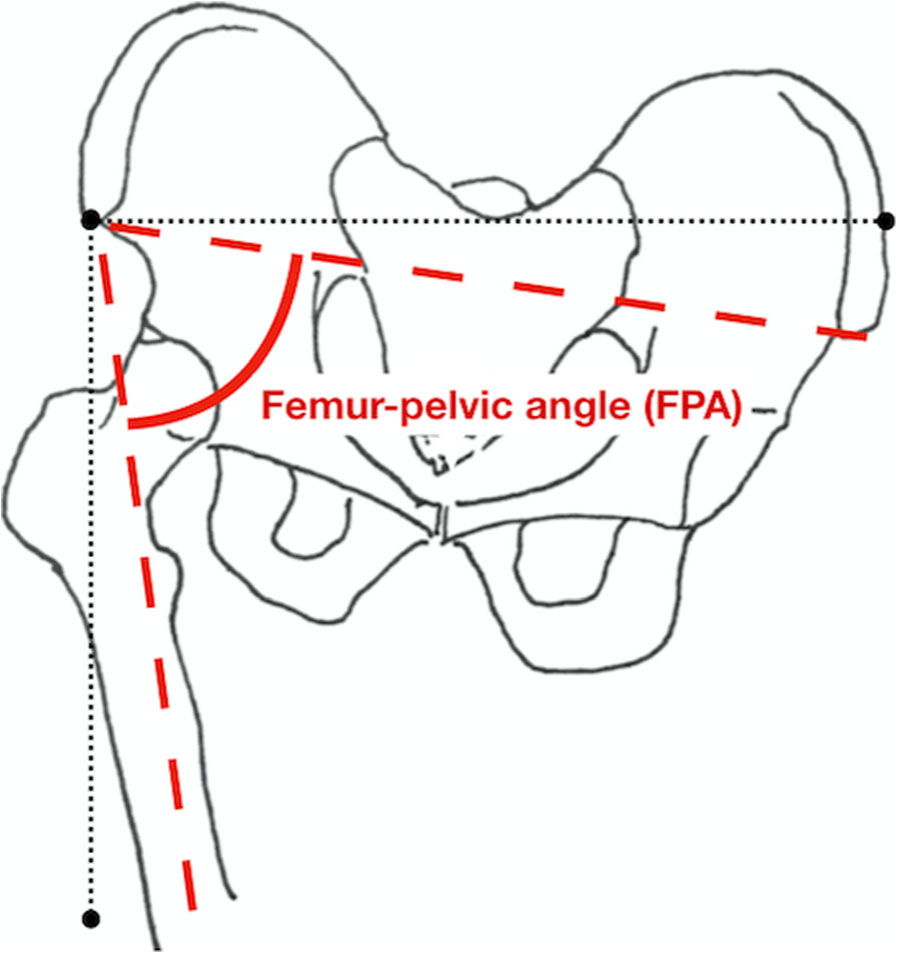
Femur-pelvic angle (FPA) measured from the still-video image in single-leg vertical drop jump (SLVDJ) test

The SLVDJ was used to investigate hip-pelvic kinematics. In the SLVDJ the player dropped off from a 10-cm box followed by a maximal vertical jump. Hip-pelvic angles were estimated from a still video image by an investigator using Java-based software (ImageJ, National Institutes of Health), and FPA, outlined in Fig. 1, was chosen for risk factor analysis. The FPA measured in a similar, but not identical 2D single-leg landing task has shown good correlation with 3D measurements [31]. Using the same methods as this study, Stensrud et al. observed moderate to excellent reliability when they measured lower extremity kinematics during the SLVDJ (ICC range = 0.58–0.89) [26].

The VDJ was used to investigate the vGRF during landing. During a valid VDJ test the player stood on the 30-cm box, dropped off the box and immediately after landing the player performed a maximal vertical jump. Absolute and weight adjusted peak vGRF and side-to-side asymmetry were investigated as potential risk factors. The same methodology has been used previously by, for example, Nilstad et al., Mok et al. and Krosshaug et al. [27–29]. They also demonstrated good to excellent reliability for peak vGRF measure in athletes (ICC range = 0.60–0.91) [28, 29].

### Injury and sport exposure registration

The primary outcomes were traumatic and non-traumatic LBP. LBP was defined as pain in the lower back area that prevented the player from taking full part in team practices and games for at least 24 h. LBP that resulted from a specific identifiable event, such as falling, was referred to as acute traumatic LBP. Non-traumatic LBP had gradual onset, without an identifiable event of trauma. Acute traumatic LBP events were categorised as “contact”, “indirect contact”, and “non-contact” [32]. A contact injury was defined as an injury sustained by the injured body region because of direct contact with another player or object and were excluded from this investigation. An indirect contact and non-contact injury were defined as occurring without direct contact to the injured body region.

Once a week one of the two study physicians contacted the teams to interview the injured players. A structured injury questionnaire (Supplementary Table 1) was used to register the injury/pain location, cause, type, time of onset and the suspected mechanism (acute traumatic vs. non-traumatic gradual onset) based on recommendations of Fuller et al. [33]. During the follow-up, the coaches collected all hours in games and team practices for each player on a monthly basis. Individual practice performed outside the scheduled team events was not included in the exposure data.

### Statistical methods

IBM SPSS Statistics (v. 23–24.0) and Chi-square test and the t-test (Mann-Whitney test when appropriate) were used for descriptive statistical analyses and the results were reported as the mean and standard deviation (SD). Cox’s proportional hazard models with mixed-effects were used to investigate the associations between potential risk factors and LBP (yes/no). This method accounts for the sports exposure and variance in follow-up time between the players. Mixed effects were used to account for the sports club as a random effect. Time-dependent variables were used, when possible, due to the tendency of changes in investigated variables over time. The individual game and practice hours from the start of the follow-up until the first event (LBP) or the end of follow-up (if no event) were included in analyses. For players reporting more than one LBP after the baseline, only the first was included. Data from all eligible players entering the follow-up were included in the analyses for the time they participated.

R (v 3.1.2; R Foundation for Statistical Computing [34]) package coxme [35] was used for the risk factor analyses. Univariate analyses were followed by multivariable analyses, where the number of adjusting variables was dependent on the number of events (10 per variable) included in the analysis, as recommended by Peduzzi et al. [36, 37]. The adjusting variables were selected from the following factors: age, sex, BMI, nicotine use, leg dominance, family history of LBP, and history of LBP. Leg dominance was used as a two-category variable: the categories ‘left’ and ‘right‘ were merged into ‘unilateral leg dominance’ and the category ‘don’t know/both’ into ‘bi-lateral/unknown leg dominance’. The adjusting factors were selected by dropping factors from the model one by one, based on their statistical significance. Only nicotine use, a history of LBP and leg dominance showed a statistically significant association with LBP. The analyses were performed using continuous and dichotomized variables. Variables were dichotomized into ‘high’ and ‘low’ using the median. The results are presented as hazard ratios (HR), 95% CIs and p-values. The player was considered as the unit of analysis, but in unilateral tasks the right and left sides were investigated separately.

## Results

**Fig. 2:**
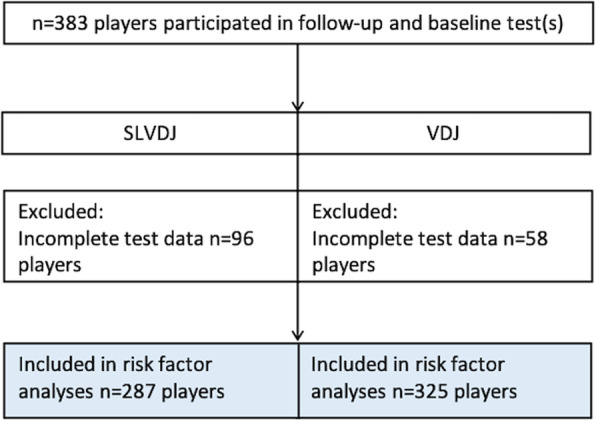
Study participant flow

Nine teams of both sports agreed to participate (Fig. 2), with a mean follow-up time of 16.5 months (range 1 to 36 months). Player demographics and baseline test results from each study year are presented in Table 2. There were some differences between the players included and excluded from the tests (Supplementary Table 2). For example, more male players and heavier players were excluded from the SLVDJ test due to ongoing injuries and for not having a valid test result. The players excluded from the VDJ test were older and heavier than those that were included.

**Table 2:** Baseline characteristics, baseline test results, and practice and game exposure during the follow up for players with and without LBP

	LBP during follow-up	Study year 2011–2012				Study year 2012–2013				Study year 2013–2014			
		n	Mean	SD	***P*** value	n	Mean	SD	***P*** value	n	Mean	SD	***P*** value
Age, years	No	106	**16.2**	**1.7**	**0.009**	138	16.7	2.0	0.659	266	16.1	2.0	0.109
	Yes	6	18.0	1.5		10	16.3	2.2		32	15.5	2.0	
Sex, %		100	20.3	5.2	0.743	128	19.3	4.4	0.569	228	**18.8**	**3.9**	**0.043**
Female	No	80	95.2			85	92.4			107	88.4		
	Yes	4	4.8			7	7.6			14	11.6		
Male	No	26	92.9			53	94.6			159	89.8		
	Yes	2	7.1			3	5.4			18	10.2		
Height, cm	No	106	170.4	8.5	0.138	138	173.2	9.1	0.609	259	174.2	9.4	0.929
	Yes	6	175.8	10.9		10	171.7	10.1		32	174.1	7.7	
Weight, kg	No	106	63.8	9.8	0.646	138	66.9	10.5	0.124	259	66.3	11.0	0.598
	Yes	6	67.3	12.4		10	62.1	8.4		32	65.1	9.0	
BMI	No	106	21.9	2.6	0.776	138	22.2	2.6	0.140	259	21.8	2.8	0.633
	Yes	6	21.6	2.0		10	21.0	1.6		32	21.4	2.1	
Sport, %													
Basketball	No	58	96.7		0.307	58	96.7		0.717	133	88.1		0.504
	Yes	2	66.7			2	3.3			18	11.9		
Floorball	No	48	92.3			80	90.9			133	90.5		
	Yes	4	7.7			8	9.1			14	9.5		
Nicotine use, %													
No	No	103	94.5		0.676	134	93.7		0.230	257	89.9		0.103
	Yes	6	5.5			9	6.3			29	10.1		
Yes	No	3	100.0			4	80.0			9	75.0		
	Yes	0	0.0			1	20.0			3	25.0		
Peak vGRF, N/kg	No	100	20.3	5.2	0.743	128	19.3	4.4	0.569	228	**18.8**	**3.9**	**0.043**
	Yes	5	19.7	4.2		6	18.0	3.7		29	**20.2**	**4.2**	
Absolute Peak vGRF, N	No	100	1289.1	392.3	0.774	128	1269.1	357.4	0.224	228	1216.7	321.3	0.122
	Yes	5	1316.5	345.5		6	1077.4	118.5		29	1298.2	308.7	
Peak vGRF asymmetry, N/kg	No	100	2.5	2.0	0.584	128	2.0	1.8	0.464	228	2.1	1.8	0.641
	Yes	5	2.1	2.0		6	2.4	1.8		29	2.1	2.0	
Absolute Peak vGRF asymmetry, N	No	100	161.2	131.6	0.662	128	131.1	123.4	0.572	228	133.6	119.4	0.735
	Yes	5	143.5	144.3		6	143.1	95.9		29	132.9	125.8	
Left leg femur-pelvic angle, degrees	No	85	80.9	4.5	0.375	103	79.9	4.4	0.303	202	80.5	5.0	0.740
	Yes	6	78.9	5.4		7	79.1	7.2		20	80.1	5.3	
Right leg femur-pelvic angle, degrees	No	91	77.5	4.9	0.905	95	76.9	4.5	0.587	199	**77.1**	**4.7**	**0.033**
	Yes	6	77.6	5.4		7	75.6	5.4		22	**74.6**	**4.9**	
Team practice hours during the follow-up, mean hours	No	106	238.1	104.5	0.341	138	201.1	89.4	0.356	266	229.0	114.6	0.923
	Yes	6	279.6	77.7		10	227.8	60.2		32	227.0	101.5	
Game hours during the follow-up, mean hours	No	106	7.1	6.0	0.597	138	8.4	5.8	0.247	266	9.1	6.0	0.240
	Yes	6	5.8	4.8		10	10.6	4.8		32	7.8	4.4	

*vGRF* vertical ground reaction force, *N* newton, *cm* centimetres, *kg* kilograms, *LBP* low back pain, *SD* standard deviation

Statistically significant results are indicated with **bold**

During the follow-up, altogether 566 athlete-years were recorded. Fifty-four percent of players (*n* = 205) reported no history of LBP at baseline. Of the 383 players, 13% (*n* = 48) sustained LBP during the follow up, 35% of them (*n* = 17) had not had back pain prior to the study. Half of the players developing LBP during the follow-up were females (52%, *n* = 25). Fifty-four percent of floorball players and 46% of basketball players had LBP during the follow-up. Most of the players who developed back pain during the follow up did so during their first follow-up year (81%) and only one player was followed for 3 years before developing LBP. LBP incidence was addressed in a previous publication [1].

### Risk factor analyses

Our results showed that the players who had a smaller FPA during SLVDJ when landing on their right leg were at increased risk for all LBP and for gradual onset non-traumatic LBP (Table 3). The analysis using dichotomous risk factors showed that players with 80° FPA or less during right leg landing, had 2.2 times higher risk for LBP during the follow-up, than players with more than 80° FPA. There was no statistically significant association between risk for LBP and FPA during left leg landing from the SLVDJ.

**Table 3:** Cox regression analysis results for femur-pelvic angle (FPA) during single-leg vertical drop jump

Continuous variables^**b,c**^	Univariate			Adjusted		
	HR	95% CI	***P*** value	HR	95% CI	***P*** value
**All LBP**						
Femur-pelvic angle, left side (^a^)	1.04	(0.97 to 1.11)	0.240	1.04	(0.97 to 1.11)	0.310
Femur-pelvic angle, right side (^a^)	**1.09**	**(1.02 to 1.17)**	**0.011**	**1.09**	**(1.02 to 1.17)**	**0.014**
**Gradual onset non-traumatic LBP**						
Femur-pelvic angle, left side (^a^)	1.04	(0.96 to 1.11)	0.370	1.03	(0.95 to 1.11)	0.480
Femur-pelvic angle, right side (^a^)	**1.10**	**(1.02 to 1.18)**	**0.013**	**1.09**	**(1.01 to 1.18)**	**0.021**
**Dichotomous variables**^d^						
**All LBP**						
Femur-pelvic angle, left side (low vs high)	1.80	(0.91 to 3.57)	0.094	1.86	(0.94 to 3.71)	0.076
Femur-pelvic angle, right side (low vs high)	**2.15**	**(1.10 to 4.21)**	**0.026**	**2.19**	**(1.12 to 4.30)**	**0.023**
**Gradual onset non-traumatic LBP**						
Femur-pelvic angle, left side (low vs high)	1.72	(0.79 to 3.73)	0.170	1.80	(0.83 to 3.90)	0.140
Femur-pelvic angle, right side (low vs high)	**2.25**	**(1.07 to 4.72)**	**0.033**	**2.30**	**(1.09 to 4.84)**	**0.028**

*HR* Hazard ratio, *CI* confidence interval, *all LBP* acute traumatic and gradual non-traumatic low back pain

^a^ degrees

^b^Adjusted with history of LBP, leg dominance

^c^ HR calculated per one-degree decrease

^d^Adjusted with history of LBP

Femur-pelvic angle, left side high > = 80.0°, low< 80.0°

Femur-pelvic angle, right side high > = 76.3°, low< 76.3°

Statistically significant results are indicated with **bold**

In the third study year, mean peak vGRF was significantly higher in players who developed LBP during the follow-up (20.2 vs. 18.8 N/kg, p-value 0.033), but no significant differences were observed between previous study years (Table 2). The Cox risk factor analyses showed no association between peak vGRF measures and LBP incidence in young floorball and basketball players (Table 4).

**Table 4:** Association between peak vGRF measures and injury risk for all LBP and gradual onset non-traumatic LBP

	Univariate		Adjusted	
	HR 95% CI	***P*** value	HR95% CI	***P*** value
**Continuous variables**^**a**^				
**All LBP**				
Peak vGRF, N/Kg	1.03 (0.97 to 1.11)	0.340	1.03 (0.96 to 1.11)	0.380
Absolute Peak vGRF, N	1.00 (1.00 to 1.00)	0.760	1.00 (1.00 to 1.00)	0.870
Peak vGRF asymmetry, N/Kg	1.00 (0.85 to 1.18)	0.990	1.00 (0.85 to 1.18)	0.990
Absolute Peak vGRF asymmetry, N	1.00 (1.00 to 1.00)	0.970	1.00 (1.00 to 1.00)	0.940
**Gradual onset non-traumatic LBP**				
Peak vGRF, N/Kg	1.00 (1.00 to 1.00)	0.610	1.00 (1.00 to 1.00)	0.690
Absolute Peak vGRF, N	1.04 (0.96 to 1.12)	0.370	1.03 (0.96 to 1.12)	0.420
Peak vGRF asymmetry, N/Kg	1.03 (0.87 to 1.23)	0.720	1.02 (0.86 to 1.22)	0.810
Absolute Peak vGRF asymmetry, N	1.00 (1.00 to 1.00)	0.710	1.00 (1.00 to 1.00)	0.790
**Dichotomous variables**^b^**(high vs. low)**				
**All LBP**				
Peak vGRF, N/Kg	1.92 (1.00 to 3.68)	0.051	1.83 (0.95 to 3.51)	0.070
Absolute Peak vGRF, N	0.99 (0.53 to 1.83)	0.960	0.94 (0.51 to 1.76)	0.860
Peak vGRF asymmetry, N/Kg	1.23 (0.66 to 2.30)	0.510	1.22 (0.65 to 2.27)	0.530
Absolute Peak vGRF asymmetry, N	1.21 (0.65 to 2.25)	0.550	1.20 (0.64 to 2.23)	0.580
**Gradual onset non-traumatic LBP**				
Peak vGRF, N/Kg	1.47 (0.73 to 2.98)	0.610	1.41 (0.69 to 2.86)	0.340
Absolute Peak vGRF, N	0.98 (0.49 to 1.97)	0.370	0.94 (0.47 to 1.88)	0.850
Peak vGRF asymmetry, N/Kg	1.31 (0.65 to 2.64)	0.720	1.30 (0.64 to 2.61)	0.470
Absolute Peak vGRF asymmetry, N	1.28 (0.64 to 2.58)	0.710	1.26 (0.63 to 2.54)	0.510

*HR* Hazard ratio, *CI* confidence interval, *LBP* low back pain, *vGRF* vertical ground reaction force, *N* Newton;

^a^Adjusted with history of LBP, leg dominance and nicotine use

^b^ All LBP: Adjusted with history of LBP and leg dominance. Gradual onset LBP: Adjusted with history of LBP

Peak vGRF high > = 18.5, low< 18.5

Absolute Peak vGRF high > = 1191.0, low< 1191.0

Peak vGRF asymmetry high > = 1.6, low< 1.6

Absolute Peak vGRF asymmetry high > = 103.3, low< 103.3

Statistically significant results are indicated with **bold**

## Discussion

The aim of this study was to investigate whether hip-pelvic kinematics and peak vGRF during landing tasks were associated with LBP incidence in youth floorball and basketball players. The first hypothesis was that the movement pattern, where the FPA is decreased during SLVDJ landing due to increased movement of the hip in the direction of adduction and contralateral pelvis drop might predispose for LBP. The second hypothesis was that players with higher or asymmetric peak vGRF during VDJ landing are at increased risk for LBP. Contrary to our second hypothesis, we did not find a statistically significant association between LBP and peak vGRF. However, our results suggested that there is an association between hip-pelvic kinematics and LBP.

The lumbo-pelvic function is an essential part of successful athletic performance [10]. According to a conceptual framework of the kinetic chain [38], a decreased or increased movement somewhere in the kinetic chain is compensated for elsewhere along the chain. This has also been suggested by Garci et al. (2015), who observed that a change in frontal plane knee kinematics resulted in changes higher in the kinetic chain [39]. It has also been shown that stability in inferior segments, such as the lower leg, is significantly correlated with superior segments, such as pelvis and back, and therefore trunk stability may be dependent on the stability of lower segments [40]. Thus, based on the kinetic chain theory it could be hypothesised that the decreased FPA may result in movement compensations and increased load and strain up and down the kinetic chain, that is in the lumbo-pelvic area as well as in the knee and lower leg. The association of trunk, pelvis and hip kinematics in relation to lower extremity complaints has been discussed [41] and previous research suggests that dysfunction distal to the injury site can be associated with future injury occurrence [42, 43].

Our results showed a small increase in risk (8%) for LBP with a one-degree decrease in the right leg FPA during the SLVDJ landing. This means a 2.2-fold increase in risk in players with less than 80° FPA during the right leg landing, compared to the players with more than 80° FPA. However, no association was detected between the left leg FPA and the risk of LBP. The difference between the right and left leg results might be due to the test procedure where the starting leg was not randomized, that is, the test was always started with the right leg. Another explanation may be the fact that in most players the right leg was their dominant (kicking) leg and the left leg was their supporting leg. This may explain why the left side was more stable during the SLVDJ. Our results are in line with previous studies suggesting that hip-pelvic kinematics are associated with injuries in athletes [11, 44, 45]. For example, findings from Bayne et al. indicated that increased knee valgus and hip adduction movements might result in increased repetitive compensatory movements from the pelvis and trunk [45]. Frontal hip-pelvic kinematics have been linked with trunk kinematics, for example increased trunk lateral lean, during single-leg tasks [46]. Gluteal muscle dysfunction has been associated with LBP [47], and it could be speculated that gluteal muscle dysfunction could result in inability to control the movement of the hip-pelvic complex during single-leg landing. In addition, the hip-pelvic movement pattern observed in this study might also be a compensatory movement resulting from several other factors, such as decreased control of the trunk over the pelvis or even control of the ankle. Therefore, in future studies, it is important to study the kinematics of the entire kinetic chain and not just a part of it.

Our second hypothesis was that vGRFs that affect the lumbar spine [19] could potentially predispose for back pain. However, to our knowledge the association between peak vGRF and LBP incidence in youth athletes has not been studied previously. According to our findings, there was no association between LBP incidence and peak vGRF or vGRF side-to-side asymmetry, measured in VDJ landing. In a cross-sectional investigation, Müller et al. were also unable to find a difference in vGRFs of youth athletes with and without LBP [48]. Future studies should investigate if loading rate is associated with LBP, because it has been shown to be a stronger risk factor for lower extremity injuries than peak GRF [49].

### Methodological considerations

The strengths of this study were the prospective design and the methods of LBP and playing exposure registrations. In addition, the sample size was relatively large. The length of follow-up varied across the sample and therefore we used Cox regression analysis. Cox regression analysis can adjust for variations in the amount of sport participation (follow-up time). Yet, due to the relatively low number of LBP events, we were unable to stratify the analyses by sex. However, it seemed that sex was not significantly associated with LBP in this sample.

Risk factors can change over time and therefore we used time-varying variables in the Cox analysis, when possible. In addition, over half (54.5%) of the players had a history of LBP at the beginning of the study and 35% (*n* = 17) of the LBP recorded during follow-up was first-time LBP. We compensated for this by adjusting the risk factor analyses with a history of LBP.

We should not overlook the fact that up to 25% of all players participating (*n* = 383) were not included in the risk factor analyses. In the SLVDJ 25% of the players and in the VDJ 19% of the players had incomplete baseline test data. The absence of these players might affect the results of this study. We are also unaware whether players refusing to participate differ from our sample. Another limitation is that we did not test the reliability of the selected tests during this study. However, the reliability of vGRF measurements has been demonstrated previously by Krosshaug and Mok and their colleagues [28, 29]. Herrington and others demonstrated in a similar test that frontal plane FPA is a reliable measurement [31]. One limitation is that in the SLVDJ test the starting leg was not randomized. The players performed the test first with the right leg and this might have had an effect on the results. When performing the test with the left leg, the players were more experienced.

The aetiology of LBP has been shown to be multifactorial [50], meaning that, in addition to external loading, internal loading such as psychosocial stress should also be recorded. The latter has been associated with the risk of sports injuries in general [51]. There are also several other risk factors that should be taken into account, such as trunk muscle symmetry [52], in addition to acknowledging the fact that risk factors are dynamic in nature and change over time [53].

## Conclusions

Our results suggested that there is an association between hip-pelvic kinematics and LBP, as measured in this study. However, we did not find a statistically significant association between LBP peak vGRF or side-to-side asymmetry of vGRF during VDJ landing. In the future, the association between hip-pelvic kinematics and LBP incidence should be investigated further to verify the results from this study.

## Supplementary information

**Additional file 1 Supplementary Table 1**. Data collected in the structured injury questionnaire.

**Additional file 2 Supplementary Table 2**. Differences between players with and without baseline test result.

## Data Availability

An anonymized form of the data can be made available from the corresponding author upon reasonable request.

## Abbreviations

LBP: Low back pain
CI: Confidence interval
SLVDJ: Single-leg vertical drop jump
VDJ: Vertical drop jump
HR: Hazards ratio
FPA: Femur-pelvic angle
vGRF: Vertical ground reaction force
GRFs: Ground reaction forces
2D: Two-dimensional
3D: Three-dimensional
SD: Standard deviation
N: Newton
Kg: Kilograms
