# Supplementary material for: Performance in dynamic movement tasks and occurrence of low back pain in youth floorball and basketball players
Source: BMC Musculoskelet Disord. 2020 Jun 5;21:350. doi: 10.1186/s12891-020-03376-1 (PMC7275454; doi:10.1186/s12891-020-03376-1)
Supplement: Supplementary file 1 — Additional file 1 Supplementary Table 1. Data collected in the structured injury questionnaire. [file 12891_2020_3376_MOESM1_ESM.docx]

**Table 1.** Data collected in the structured injury questionnaire^#^

| 1. Date of injury 2. Where did the injury occur? (in official game / friendly game / sports specific training / conditioning training / other)    - Questions for game injury:      - Playing position      - Game period      - Time of game period 3. Surface (wooden / artificial / other, specify) 4. Injured body part 5. Injured body side (right / left / both / not applicable) 6. Type of injury 7. Onset of injury (acute / overuse) 8. New / recurrent injury    - Question for recurrent injury:  Specify date of return to full participation from the previous injury. 9. Use of protective or supportive equipment (no/yes, specify) 10. Was the injury caused by contact or collision? (no / yes, contact with another player / yes, contact with the ball, stick or other object)     - Question for contact injury: Direct contact to the injured body part / indirect contact. 11. Describe the injury situation 12. Existing video material of the injury situation (no / yes) 13. Where the injury was treated 14. Medical investigations (MRI / ultrasound / other, specify) 15. Diagnosis 16. Orthopedic operations due to the injury (no / yes, specify) 17. Time-loss from training (number of days) 18. Time-loss from games (number of games) 19. Time-loss from school/work (number of days) 20. Previous menstruation (date) 21. Direct costs of the injury |
| --- |

^#^ Structured injury questionnaire was used by the study physician, when interviewing the injured players
