# Supplementary material for: Performance in dynamic movement tasks and occurrence of low back pain in youth floorball and basketball players
Source: BMC Musculoskelet Disord. 2020 Jun 5;21:350. doi: 10.1186/s12891-020-03376-1 (PMC7275454; doi:10.1186/s12891-020-03376-1)
Supplement: Supplementary file 2 — Additional file 2 Supplementary Table 2. Differences between players with and without baseline test result. [file 12891_2020_3376_MOESM2_ESM.docx]

**SUPPLEMENTARY TABLE 2.** Differences between players with and without baseline test result

|  | Valid result from test | Study year 2011-2012 | | | | Study year 2012-2013 | | | | Study year 2013-2014 | | | |
| --- | --- | --- | --- | --- | --- | --- | --- | --- | --- | --- | --- | --- | --- |
| Single-leg Vertical drop jump |  | n | Mean | SD | P value | n | Mean | SD | P value | n | Mean | SD | P value |
| Age. years | No | 7 | 17.4 | 1.8 | 0.104 | 24 | 17.0 | 1.9 | 0.332 | 56 | 15.9 | 2.0 | 0.501 |
|  | Yes | 105 | 16.2 | 1.7 |  | 124 | 16.6 | 2.0 |  | 242 | 16.0 | 2.0 |  |
| Height. cm | No | 7 | 172.5 | 7.8 | 0.493 | 24 | 174.9 | 8.8 | 0.243 | 54 | 173.1 | 8.7 | 0.482 |
|  | Yes | 105 | 170.6 | 8.8 |  | 124 | 172.8 | 9.2 |  | 237 | 174.5 | 9.3 |  |
| Weight. kg | No | 7 | 72.4 | 13.7 | 0.079 | 24 | 75.2 | 10.8 | ≤0.001 | 54 | 71.2 | 12.2 | 0.002 |
|  | Yes | 105 | 63.4 | 9.5 |  | 124 | 65.0 | 9.6 |  | 237 | 65.0 | 10.1 |  |
| BMI | No | 7 | 24.3 | 3.9 | 0.089 | 24 | 24.5 | 2.6 | ≤0.001 | 54 | 23.7 | 3.4 | ≤0.001 |
|  | Yes | 105 | 21.8 | 2.4 |  | 124 | 21.7 | 2.3 |  | 237 | 21.3 | 2.4 |  |
| Team practice hours | No | 7 | 185.1 | 121.1 | 0.188 | 24 | 185.9 | 95.0 | 0.463 | 56 | 197.3 | 134.9 | 0.016 |
|  | Yes | 105 | 244.0 | 101.7 |  | 124 | 206.2 | 86.5 |  | 242 | 236.1 | 106.5 |  |
| Game hours | No | 7 | 5.0 | 4.8 | 0.315 | 24 | 7.1 | 6.4 | 0.117 | 56 | 7.6 | 5.4 | 0.076 |
|  | Yes | 105 | 7.2 | 6.0 |  | 124 | 8.8 | 5.6 |  | 242 | 9.3 | 5.9 |  |
| Sport. % |  |  |  |  |  |  |  |  |  |  |  |  |  |
| Basketball | No | 4 | 6.7 |  | 0.845 | 6 | 10.0 |  | 0.090 | 30 | 19.9 |  | 0.630 |
|  | Yes | 56 | 93.3 |  |  | 54 | 90.0 |  |  | 121 | 80.1 |  |  |
| Floorball | No | 3 | 5.8 |  |  | 18 | 20.5 |  |  | 26 | 17.7 |  |  |
|  | Yes | 49 | 94.2 |  |  | 70 | 79.5 |  |  | 121 | 82.3 |  |  |
| Nicotine use. % |  |  |  |  |  |  |  |  |  |  |  |  |  |
| No | No | 7 | 6.4 |  | 0.650 | 22 | 15.4 |  | 0.142 | 53 | 18.5 |  | 0.574 |
|  | Yes | 102 | 93.6 |  |  | 121 | 84.6 |  |  | 233 | 81.5 |  |  |
| Yes | No | 0 | 0.0 |  |  | 2 | 40.0 |  |  | 3 | 25.0 |  |  |
|  | Yes | 3 | 100.0 |  |  | 3 | 60.0 |  |  | 9 | 75.0 |  |  |
| Sex. % |  |  |  |  |  |  |  |  |  |  |  |  |  |
| Female | No | 3 | 3.6 |  | 0.043 | 9 | 9.8 |  | 0.006 | 21 | 17.4 |  | 0.600 |
|  | Yes | 81 | 96.4 |  |  | 83 | 90.2 |  |  | 100 | 82.6 |  |  |
| Male | No | 4 | 14.3 |  |  | 15 | 26.8 |  |  | 35 | 19.8 |  |  |
|  | Yes | 24 | 85.7 |  |  | 41 | 73.2 |  |  | 142 | 80.2 |  |  |
| 3D Vertical drop jump |  |  |  |  |  |  |  |  |  |  |  |  |  |
| Age. years | No | 15 | 15.9 | 2.1 | 0.197 | 90 | 17.0 | 1.9 | 0.009 | 128 | 17.1 | 2.0 | ≤0.001 |
|  | Yes | 97 | 16.4 | 1.7 |  | 58 | 16.1 | 2.0 |  | 170 | 15.2 | 1.6 |  |
| Height. cm | No | 15 | 175.5 | 9.1 | 0.033 | 90 | 171.8 | 9.2 | 0.019 | 121 | 174.2 | 9.4 | 0.823 |
|  | Yes | 97 | 170.0 | 8.4 |  | 58 | 175.2 | 8.9 |  | 170 | 174.2 | 9.1 |  |
| Weight. kg | No | 15 | 71.1 | 12.7 | 0.008 | 90 | 65.9 | 11.0 | 0.158 | 121 | 68.8 | 10.2 | ≤0.001 |
|  | Yes | 97 | 62.9 | 9.1 |  | 58 | 67.7 | 9.5 |  | 170 | 64.3 | 10.8 |  |
| BMI | No | 15 | 23.0 | 3.4 | 0.233 | 90 | 22.3 | 2.7 | 0.453 | 121 | 22.6 | 2.7 | ≤0.001 |
|  | Yes | 97 | 21.7 | 2.4 |  | 58 | 22.0 | 2.4 |  | 170 | 21.1 | 2.6 |  |
| Team practice hours ^A^ | No | 15 | 204.1 | 114.8 | 0.134 | 90 | 204.5 | 89.5 | 0.716 | 128 | 216.0 | 113.0 | 0.102 |
|  | Yes | 97 | 245.9 | 101.0 |  | 58 | 200.4 | 86.0 |  | 170 | 238.5 | 112.6 |  |
| Game hours ^B^ | No | 15 | 5.6 | 4.4 | 0.442 | 90 | 9.1 | 6.2 | 0.149 | 128 | 8.6 | 5.5 | 0.482 |
|  | Yes | 97 | 7.3 | 6.1 |  | 58 | 7.6 | 4.8 |  | 170 | 9.2 | 6.0 |  |
| Sport, % |  |  |  |  |  |  |  |  |  |  |  |  |  |
| Basketball | No | 6 | 7.1 |  | 0.001 | 64 | 69.6 |  | 0.005 | 59 | 48.8 |  | 0.094 |
|  | Yes | 78 | 92.9 |  |  | 28 | 30.4 |  |  | 62 | 51.2 |  |  |
| Floorball | No | 9 | 32.1 |  |  | 26 | 46.4 |  |  | 69 | 39.0 |  |  |
|  | Yes | 19 | 67.9 |  |  | 30 | 53.6 |  |  | 108 | 61.0 |  |  |
| Nicotine use, % |  |  |  |  |  |  |  |  |  |  |  |  |  |
| No | No | 11 | 18.3 |  | 0.099 | 35 | 58.3 |  | 0.610 | 48 | 31.8 |  | 0.000 |
|  | Yes | 49 | 81.7 |  |  | 25 | 41.7 |  |  | 103 | 68.2 |  |  |
| Yes | No | 4 | 7.7 |  |  | 55 | 62.5 |  |  | 80 | 54.4 |  |  |
|  | Yes | 48 | 92.3 |  |  | 33 | 37.5 |  |  | 67 | 45.6 |  |  |
| Sex, % |  |  |  |  |  |  |  |  |  |  |  |  |  |
| Female | No | 15 | 13.8 |  | 0.490 | 86 | 60.1 |  | 0.371 | 122 | 42.7 |  | 0.615 |
|  | Yes | 94 | 86.2 |  |  | 57 | 39.9 |  |  | 164 | 57.3 |  |  |
| Male | No | 0 | 0.0 |  |  | 4 | 80.0 |  |  | 6 | 50.0 |  |  |
|  | Yes | 3 | 100.0 |  |  | 1 | 20.0 |  |  | 6 | 50.0 |  |  |
| vGRF, vertical ground reaction force; N, newton; cm, centimetres; kg, kilograms, LBP, low back pain; SD, standard deviation; | | | | | | | | | | | | | |
